# Optimization of HS-SPME-GC-MS for the Determination of Volatile Flavor Compounds in Ningxiang Pork

**DOI:** 10.3390/foods12020297

**Published:** 2023-01-08

**Authors:** Hu Gao, Fang Yang, Bangqiang Zhu, Shishu Yin, Yawei Fu, Yiyang Li, Yinchang Liao, Meng Kang, Yuebo Zhang, Jun He, Yulong Yin, Kang Xu

**Affiliations:** 1 Animal Nutrition Genome and Germplasm Innovation Research Center and Hunan Provincial Key Laboratory for Genetic Improvement of Domestic Animal, College of Animal Science and Technology, Hunan Agricultural University, Changsha 410128, China; 2 Guangdong Laboratory for Lingnan Modern Agriculture, Guangzhou 510642, China; 3 Laboratory of Animal Nutrition Physiology and Metabolism, The Chinese Academy of Sciences, The Institute of Subtropical Agriculture, Changsha 410125, China

**Keywords:** HS-SPME-GC-MS, Ningxiang pork, volatile flavor compounds, method optimization

## Abstract

This study attempts to explore the suitable conditions for the detection of volatile flavor compounds (VFCs) in Ningxiang pork by headspace solid-phase microextraction and gas chromatography-mass spectrometry (HS-SPME-GC-MS). Ningxiang pigs were harvested from a slaughterhouse and a longissimus dorsi sample was collected from each animal. The VFCs of Ningxiang pork can be strongly impacted by the detection conditions (columns, weight of meat samples, heat treatment time, equilibrium conditions, and extraction conditions) that need to be optimized. Our results also provided the optimal test conditions: weighing 5 g of meat samples, grinding for 30 s in a homogenizer, heat treatment at 100 °C for 30 min, equilibration at 70 °C for 30 min, and extraction at 100 °C for 50 min. Furthermore, the feasibility and representativeness of the test method were confirmed based on principal component analysis and a comparison of the three pork VFCs. These findings offer researchers a unified and efficient pretreatment strategy to research pork VFCs.

## 1. Introduction

Volatile flavor compounds (VFCs) of meat are produced during the cooking process by a series of chemical reactions of meat flavor precursor substances [1]. Meat flavor is a broad sense of food stimulation produced by the human senses of smell and taste and the trigeminal nerve [2]. Meat flavor precursors serve as the foundation for flavor formation. Water-soluble flavor precursors primarily produce VFCs via the Maillard reaction and thermal degradation of thiamine, both of which significantly contribute to meat aroma [3,4,5]. Lipids primarily generate VFCs through lipid oxidative degradation reactions, which are the key substances in the formation of meat aroma [6,7,8], the majority of which are carbonyl compounds and ester compounds produced by thermally induced lipid acyl chain oxidation reactions [9,10]. These substances are concentrated in the sensory aspect as aroma [11,12,13].

The VFCs produced by the Maillard and Streck reactions of pork have a greater contribution to the sensory evaluation of meat quality, particularly the aldehydes produced by lipids, ketones, esters, furans, and other volatile compound reactions [14,15,16]. As a typical indigenous Chinese breed of pig, the Ningxiang pig is one of China’s four famous pigs [17]. Its meat is tender and fresh, with a moderate intramuscular fat content and an excellent flavor. Intramuscular fat can significantly contribute to the flavor of meat. Pork flavor detection methods generally include sensory inspection, electronic nose, and GC-MS [18,19,20]. Human factors greatly influence sensory testing, and electronic noses can only detect a broad range of volatile compounds but cannot perform qualitative or quantitative analysis.

Headspace solid-phase microextraction and gas chromatography–mass spectrometry (HS-SPME-GC-MS) extracts target analytes using a polymer coating or adsorbent coated on quartz glass fiber as an adsorption medium. The extraction effect was primarily influenced by the coating composition, thickness, length of the fiber tip, and distribution of VFCs in the headspace volume, based on the balance of VFCs in the headspace, the extraction phase, and the sample. Previous research has shown that HS-SPME-GC-MS has the characteristics of objective, accurate, and rapid qualitative and quantitative determination of volatile substances, as well as good repeatability [21]. HS-SPME-GC-MS has been widely utilized in recent years to detect VFCs in meat [19,22,23,24], wine [25,26,27], tea [28], potato [29], cheese [30], and fruit [31,32]. There are still significant discrepancies in the quantity of chemicals detected in the VFCs of water-bath, heat-treated pork by different researchers, although numerous pretreatment protocols for the detection of pig VCFs by HS-SPME-GC-MS have been described [23,33,34,35]. The detection criteria for VFCs of pork heat-treated in a water bath have not been standardized. To compare VFCs among meats, the development of a reliable and repeatable detection method is essential.

In this study, the effects of heat treatment time, equilibrium time and temperature, and extraction time and temperature on the detection of VFCs in Ningxiang pork were given based on the HS-SPME-GC-MS method. This task was accomplished by combining a single factor and orthogonal test. Additionally, the optimal pretreatment conditions for detecting pig VFCs can be determined. The VFCs of Ningxiang, Duroc, and DLY (Landrace (Yorkshire × Duroc), Ternary hybrid) pigs were detected to confirm the viability and representativeness of the experimental detection conditions. This work is important for creating a reliable and dependable VFCs detection method for pigs.

## 2. Materials and Methods

### 2.1. Animal Harvest and Sample Collection

Ningxiang pig (*n* = 35) samples were collected from the Chu Weixiang Slaughtering and Cutting Plant in Ningxiang City, Hunan Province, Duroc (*n* = 35), and DLY (*n* = 35) (Duroc × (Landrace × Yorkshire), Ternary hybrid) pork samples were obtained from the Tangrenshen Slaughtering and Cutting Plant in Zhuzhou City, Hunan Province. Pigs were shocked with electricity and then exsanguinated during the slaughter. Afterwards, 20~30 g samples of each pig’s longissimus dorsi between the sixth and eleventh ribs were taken within two hours of death, similar to our earlier investigation [20]. Then the samples were crushed by a grinder (180E-Y, Nail, Cixi, China), packed and kept at a constant temperature of −80 °C with a steady level of humidity. The pork samples were removed and defrosted for 24 h at 4 °C in a refrigerator. After the test began, the samples were removed, brought to room temperature, and heated in a water bath at 100 °C (DK-98-Ⅱ, TAISITE, Tianjin, China). The air had 70% humidity, and the room temperature was 25 °C. The Hunan Agricultural University Ethics Association examined and approved all animal experiments used in this study (approval number 2020047), and the research procedure fully complied with all applicable international ethical standards.

### 2.2. Headspace Solid-Phase Microextraction

To remove any contaminant peaks from the empty needle of a gas chromatography mass spectrometer (product model QP-2010, Shimadzu, Japan), the employed solid-phase microextraction tip was aged at 240 °C for 40 min at the injection port, and there are four kinds of coating materials for the microextraction tip, namely DVB (Divinylbenzene), CAR (Carboxen), PDMS (Polydimethylsiloxane), and CAR-PDMS (Carboxen-Polydimethylsiloxane). Then, a tissue homogenizer (T10 basic ULTRA-TURRAX^®^, IKA, Staufen, Germany) and an electronic scale (Xin Yuan Digital Scale-8006, Guangdong, China) were used to weigh 5 g of the sample. Thirty seconds of grinding was followed by placement in a 15 mL Teflon septum headspace special SPME vial, which was then heated in a 100 °C thermostatic water bath for 5 min before cooling to room temperature prior to extraction.

### 2.3. Gas Chromatography–Mass Spectrometry

Capillary conditions: first, nitrogen is the carrier gas, with a flow rate of 2 mL/min; second, the injection port’s temperature is 250 °C, without splitting; third, the initial temperature is 35 °C, which is held for 6 min; fourth, the temperature is increased to 130 °C at 4 °C/min and held for 2 min; and last, the temperature is increased to 230 °C at 8 °C/min and held for 5 min.

Mass spectrometry conditions: the ionization mode was EI, the electron energy was 70 eV, the ion source temperature was 230 °C, the interface temperature was 250 °C, the filament current was 150 A, and the data were collected in the range of 30–500 amu (amu).

### 2.4. Screening Capillary Column and Extraction Head

Screening columns: The effectiveness of the 3 columns’ detection was evaluated (HP-88-100 m, strong polarity; DB-5-30 m, weak polarity; DB-5-60 m, weak polarity). Heat treatment was conducted at 100 °C for 3 min; equilibration was achieved at 70 °C for 40 min; extraction was performed at 70 °C for 40 min; and analysis was conducted for 5 min.

Screening extraction heads: The detection performance of four different coated extraction heads was compared. The test conditions are listed in Table 1: DB-5-60 m capillary column, heat treatment at 100 °C for 30 min, equilibration at 70 °C for 40 min, extraction at 70 °C for 40 min, and analysis for 5 min.

### 2.5. Single-Factor Test

#### 2.5.1. Heat Treatment Time

The test conditions were DB-5-60 m capillary column, Gray 50/30 extraction head, heat treatment at 100 °C, equilibration at 70 °C for 40 min, extraction at 70 °C for 40 min, and analysis for 5 min. The heat treatment time gradient settings were 10 min, 20 min, 30 min, 40 min, 50 min, and 60 min.

#### 2.5.2. Equilibration Time

The test conditions were DB-5-60 m capillary column, Gray50/30 extraction head, heat treatment at 100 °C for 30 min, equilibration at 70 °C, extraction at 70 °C for 40 min, and analysis for 5 min. The equilibrium time gradient settings were 10 min, 20 min, 30 min, 40 min, 50 min, and 60 min.

#### 2.5.3. Equilibrium Temperature

The test conditions were DB-5-60 m capillary column, Gray50/30 extraction head, heat treatment at 100 °C for 30 min, equilibration for 40 min, extraction at 70 °C for 40 min, and analysis for 5 min. The equilibrium temperature gradient settings were 50 °C, 60 °C, 80 °C, and 100 °C.

#### 2.5.4. Extraction Time

The test conditions were DB-5-60 m capillary column, Gray50/30 extraction head, heat treatment at 100 °C for 30 min, equilibration at 70 °C for 40 min, extraction at 70 °C, and analysis for 5 min. The extraction time gradient settings were 10 min, 20 min, 30 min, 40 min, 50 min, and 60 min.

#### 2.5.5. Extraction Temperature

The test conditions were DB-5-60 m capillary column, Gray50/30 extraction head, heat treatment at 100 °C for 30 min, equilibration at 70 °C for 40 min, extraction for 40 min, and analysis for 5 min. The extraction temperature gradient settings were 50 °C, 60 °C, 80 °C, and 100 °C.

### 2.6. Orthogonal Test

An orthogonal experiment was created in accordance with the findings of the single-factor experiment. The experimental settings are provided in Table 2 for the C (orthogonal table format) orthogonal table. A total of 36 trials were conducted, with each experiment being performed twice.

### 2.7. Statistical Analysis

The total ion current chromatogram and experimental data of the sample were analyzed using the analysis software (Origin; Xcalibur^TM^ 4.2, ThermoFisher, Waltham, MA, USA), which matched the NIST spectral library and Wiley spectral library on the internet. The identification results with a positive and negative matching degree greater than 80 were chosen. To examine the importance of variations between two detection findings under various circumstances, variance analysis and graphing on the discovered data were performed using GraphPad Prism 8. The mixOmics package in Rstudio was selected to perform partial least squares discriminant analysis (PLS-DA) on the data from the three groups of VFCs.

## 3. Results and Discussion

### 3.1. Influencing Factors on GC-MS Analysis

#### 3.1.1. Effect of Columns on VFCs

The findings show that, for the same conditions, the nonpolar columns DB-5-30 m and DB-5-60 m detected a total of 44 peaks with a total peak area of 1.500 × 10^7^ and 67 peaks with a total peak area of 2.623 × 10^7^ each. Twenty-three peaks were identified with the powerful polar column HP-88-100 m, with a total peak area of 4.367 × 10^6^ (Table 3, Figure S1). Despite being the longest, the separation effect of the HP-88-100 m capillary column under identical pretreatment conditions is inferior to that of the DB-5-30 m nonpolar capillary column, which has a column length of 30 m and is even worse than that of DB-5-60 m. The two nonpolar columns (DB-5-30 m and DB-5-60 m) produced better separation, as evidenced by a higher number of identified peaks and a larger total peak area.

This suggests that the polarity and length of the capillary column does affect the way volatile chemicals are separated, as the majority of the volatile molecules in meat are moderate or nonpolar [36,37]. Therefore, it makes sense that nonpolar columns are preferably used for the separation and analysis of VFCs in meat, such as chicken [38], beef [39], and pork [40]. In addition, the length of the column may also influence the separation of volatile chemicals and the identification of compounds in GC-MS. Compared to the DB-5-30 m column, the nonpolar DB-5-60 m column has a superior separation impact on the VFCs of pork. So, the nonpolar DB-5-60 m column was chosen for further study.

#### 3.1.2. Effect of Extraction Head on VFCs

**Figure 1:**
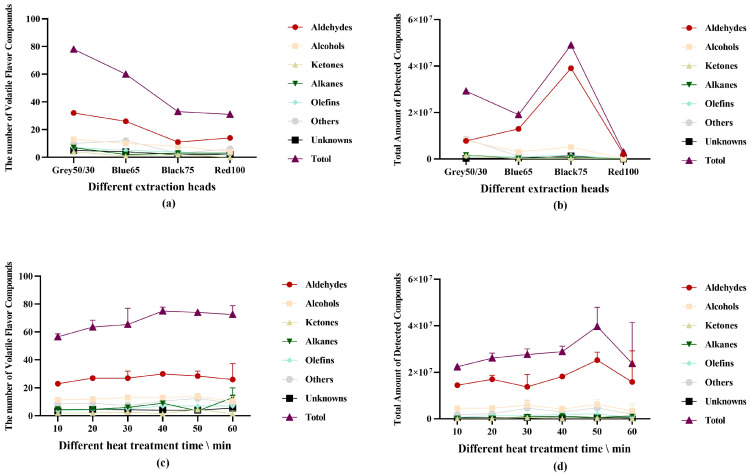
(**a**) The number of different kinds of volatile flavor compounds detected by different extraction heads; (**b**) the total amount of volatile flavor compounds detected by different extraction heads; (**c**) the number of volatile flavor compounds detected under different heat treatment time conditions; (**d**) the total amount of volatile flavor compounds detected under different heat treatment time conditions.

According to the coating material and extraction head thickness, the test results of four extraction heads were compared to identify the best extraction head for identifying VFCs in Ningxiang pork. The extraction head of the Gray50/30 model, whose coating materials included DVB, CAR, and PDMS, had the best detection effect, according to the findings of the detection of the total ion chromatogram (Figure 1a, Table 1). The number of chemicals discovered decreases with the structural richness of the coating (Figure S2). The analyte typically contains many volatile substances that the extraction head’s single structure cannot entirely adsorb. The main application scope of DVB/CAR-PDMS is volatile and semivolatile aroma substances, indicating that Ningxiang pork was rich in aroma substances after heat treatment. In addition, the PDMS coating (Red100) is mainly used to adsorb nonpolar substances in combination with CAR and DVB materials (the extraction head is Gray50/30), which significantly increases the type and quantity of adsorbed substances. The volatile compounds of Ningxiang pork after heat treatment accounted for a sizable proportion of medium polar substances. Blue65 detected significantly more substances than Black75 (Figure 1a), but the total amount of adsorbed substances was opposite (Figure 1b), probably because the volatile substances with larger molecular weight could not be adsorbed by CAR/PDMS after the heat treatment of Ningxiang pork. Additionally, the covering of Black75 is thicker than that of Blue65, which leads to more species being adsorbed.

As in previous studies on the extraction of VFCs from pork [34,41], beef [42], and mutton [43,44], the best selection of extraction head is still the Gray50/30 model made of DVB/CAR-PDMS. Furthermore, the choice of extraction head for this model is consistent with the results of J. Yang’s choice of extraction head model [37]. Therefore, the characteristics and thickness of the coating materials have a significant impact on the selectivity and enrichment of VFCs. We should select the coating materials of the traction head according to the standards of similarity and compatibility, so as to better adsorb VFCs in the sample and obtain more comprehensive data.

#### 3.1.3. Effect of Heat Treatment on VFCs

In this study, the materials underwent various levels of heat treatment. The findings demonstrated that as the heat treatment duration was extended, the ion peak appearance was delayed, the peak separation was decreased, and the separation effect of volatile chemicals was enhanced (Figure S3, Figure 1c). The total ion peak area dramatically decreased (Figure 1d) (*p* < 0.05), and the generation of chemicals in the experiment was not sufficiently steady and repeatable when the heat treatment time exceeded 50 min. There was no significant difference in the types of volatile substances in Ningxiang pork treated with different heat treatment times (*p* > 0.05), and the total amount of substances was the highest at 50 min. 

Boiling, frying, and roasting are examples of heat treatment that will directly alter the chemical reaction between two taste precursors [45]. As a method of food heat treatment, high-temperature heat treatment has the potential to partially dissolve and degrade some flavoring compounds and to accelerate the creation of new taste compounds via chemical reactions. The initial Maillard reaction will be competitively inhibited by the fatty aldehydes created by lipid oxidation if the duration is too short, and the production of flavor compounds will be reduced. These results will have an impact on the extraction effect. The Maillard reaction will produce compounds that prevent lipid oxidation if the reaction period is too long [46], and the principal flavor compounds, such as aldehydes, alcohols, and ketones, will produce esters and alkanes, which will lower the quality of the substances detected by GC-MS. In this study, the overall volatile matter and total content of Ningxiang pork decreased when heat-treated in a 100 °C water bath for more than 50 min, and the overall flavor of the meat, such as overcooked flavor [47,48,49], was reduced when heat-treated for too long. Therefore, the heat treatment time of Ningxiang pork should not exceed 50 min.

#### 3.1.4. Effect of Equilibration Conditions on VFCs

To enrich and achieve dynamic equilibrium in the upper part of the headspace bottle, which is advantageous to the extraction of volatile compounds by the extraction head in the headspace, the VFCs of pork should be balanced under specific conditions before extraction. The rate at which the volatile chemicals achieve equilibrium during this procedure will depend on the equilibration temperature and equilibration period.

**Figure 2:**
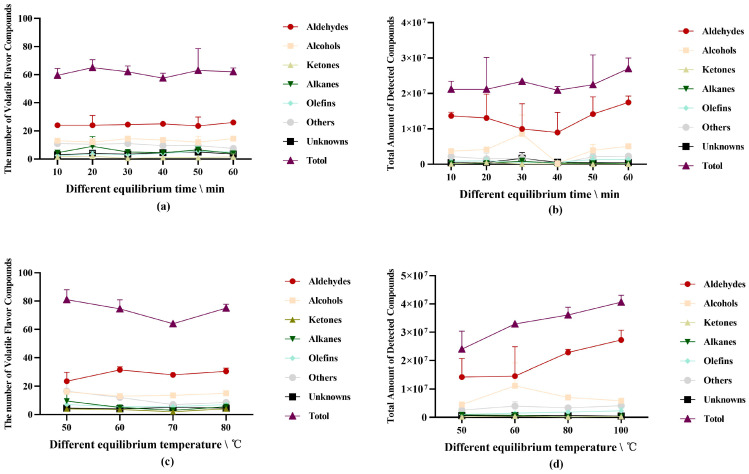
(**a**) The number of volatile flavor compounds detected under different equilibration time conditions; (**b**) the total amount of volatile flavor compounds detected under different equilibrium time conditions; (**c**) the number of volatile flavor compounds detected under different equilibrium temperature conditions; (**d**) the total amount of volatile flavor compounds detected under different equilibrium temperature conditions.

According to the findings (Figure 2a,b), the equilibrium conditions had a slight impact on the number of compounds that were detected (*p* > 0.05). However, the difference between the conditions was highly significant (*p* < 0.01) for the total amount of substances (Figure 2c,d). The total number of compounds discovered increased overall as the equilibrium duration and temperature increased, and the equilibrium amount of substances detected increased (Figures S4 and S5). The overall amount of aldehydes produced significantly increased (*p* < 0.05) as the reaction duration and equilibrium temperature increased. Additionally, as the equilibrium temperature increased, so did the production of unidentified compounds. The low-temperature oxidation of lipids produces many aldehydes [50]; hence, more aldehydes are produced when the equilibrium is brief (less than 30 min) or when the equilibrium temperature is low. The production of aldehydes rapidly increased as temperature and time increased, possibly because the Maillard reaction became stronger. The initial Maillard process was suppressed by fatty aldehydes created by lipid oxidation. However, reactive aldehydes will encourage the late reaction, leading to the production of additional novel, sulfur-containing carnivorous compounds with alkyl chains [46]. In the middle and late phases of the Maillard reaction, aldehyde compounds are involved, and while numerous additional reactive aldehydes are formed, thiophene, furan, and other substances are also produced in greater quantities. The results also illustrate the relationship between lipid oxidation and Maillard reactions; however, equilibrium conditions have different effects on various substances by affecting the reaction between flavor precursors, and aldehydes are the most affected.

Equilibrium can enhance the stability and effectiveness of the extraction process. Aldehydes, alcohols, nitrogen-containing chemicals, and hydrocarbons are primarily impacted by the equilibrium temperature. The heat-treated samples continued to react in the headspace vial during equilibration to reach dynamic equilibrium. The species of the compounds in the headspace vial do not change much in this state, but the interactions between them will cause alterations in the relative contents of the substances. In this experiment, the aldehydes dramatically increased as the equilibrium time and temperature increased, whereas the alcohols, nitrogen-containing compounds, and hydrocarbons significantly declined.

#### 3.1.5. Effect of Extraction Conditions on VFCs

The temperature of the extraction process has an impact on both the extraction rate and extraction time. The extraction head will more quickly attain adsorption equilibrium if the extraction temperature parameters are identified under which the extraction head more quickly absorbs volatile substances. Early in the extraction process, volatile chemicals are rapidly concentrated on the extraction coating, and as extraction proceeds, the rate of adsorption decreases. The adsorption of the extraction head is saturated as the equilibrium state approaches, and even increasing the extraction time has a small impact. Therefore, it is necessary to identify the best extraction conditions to improve the experimental efficiency.

**Figure 3:**
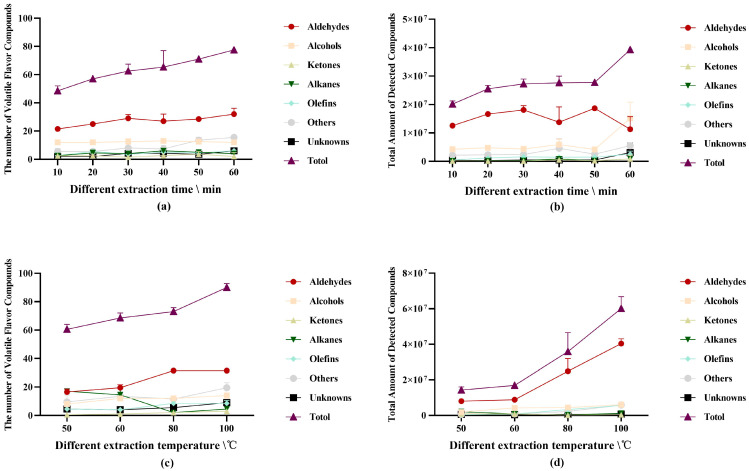
(**a**) The number of volatile flavor compounds detected under different extraction time conditions; (**b**) the total amount of volatile flavor compounds detected under different extraction time conditions; (**c**) the number of volatile flavor compounds detected under different extraction temperature conditions; (**d**) the total amount of volatile flavor compounds detected under different extraction temperature conditions.

First, Figures S6 and S7 show that the separation effect between two compounds steadily improves with an increase in extraction time and temperature. The retention time (R.time) after 30 min of substance quantity and total amount significantly increased (*p* < 0.05), as did the quantity and total amount of substances. When the extraction duration is short or the extraction temperature is low, the extraction effect is weak, resulting in poor GC-MS analysis findings due to the coating of the Gray50/30 extraction head. Second, both the total amount of substances and the number of substances that were detected significantly changed when the extraction conditions were altered (Figure 3a–d). The total amount of substances increased as the extraction time and temperature were increased, and at 100 °C, it reached its highest level (Figure 3d) and did not reach dynamic equilibrium. According to studies, there is a significant correlation among sample size, container volume, and test findings. In addition to improving repeatability, the detection threshold will rise as the sample size increases. The extraction time and temperature are no longer increased due to the effectiveness and simplicity of the test. Lower extraction temperatures result in more alkanes (Figure 3b) but lower total ion peak areas (Figure S7A,B), which means lower relative content. The relative content did not significantly vary as the extraction conditions changed (Figure 3c,d), suggesting that the newly discovered compounds included fewer alkanes. As the extraction temperature and duration increased, the number and total amount of detected unidentified compounds increased (Figure 3b,c).

Therefore, altering the extraction conditions has a direct impact on the GC-MS analysis results, and the quantity and total amount of compounds discovered are positively connected with the temperature and duration of the extraction. To further examine the distinctions between Ningxiang pork and other varieties of pigs and to identify the distinctive flavor components of Ningxiang pork, it is advantageous to alter the adsorption impact of the extraction head on unknown compounds.

### 3.2. Orthogonal Test Optimized Condition Screening

In accordance with the findings of the single-factor test, an orthogonal test was conducted to improve the HS-SPEM-GC-MS test conditions for the detection of VFCs in Ningxiang pork, and the impact of various factors on the identification of VFCs in Ningxiang pork was also discussed. In range analysis, the higher the R value is, as shown in Table 4, the greater the overall impact of this factor, offering recommendations on the level of factor to be used. The total ion chromatographic peaks of the HS-SPEM-GC-MS were considerably affected by the extraction temperature in terms of peak area. The largest impact derived the identification of the total amount of VFCs in Ningxiang pigs. The types of VFCs, particularly alcohols and aldehydes, are mostly influenced by extraction time, while the equilibrium temperature primarily affects ketones. According to the variance analysis, there was no discernible difference between the various test levels for the orthogonal test components (*p* > 0.05). Therefore, we simply took into account the test efficiency and the actual test material content while choosing the combination of test conditions.

The best test settings were chosen to produce a high-test efficiency, greater quantity, and higher concentration of the compounds detected: 5 g of meat samples were weighed, homogenized for 30 s in a homogenizer, heated for 30 min at 100 °C, equilibrated for 30 min at 70 °C, and extracted for 50 min at 100 °C.

### 3.3. Validation of the Feasibility of Exploring Experimental Conditions

**Figure 4:**
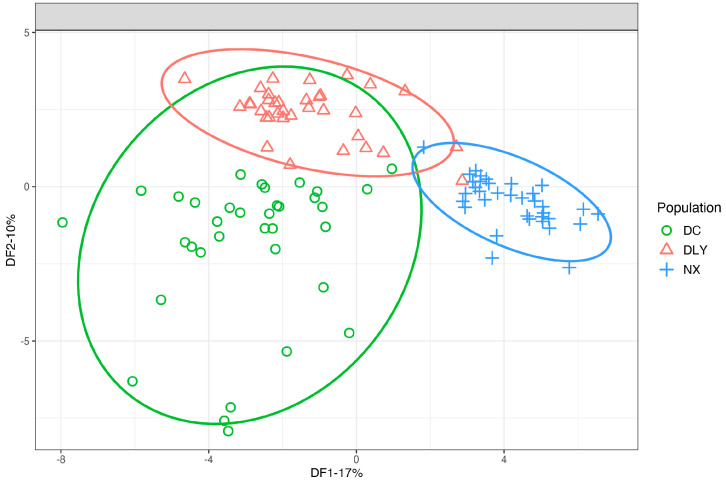
PLS-DA of volatile flavor compounds in three pork breeds.

To verify the feasibility and representativeness of the pretreatment method, we applied this method to detect VFCs in longissimus muscle from 105 pigs (35 individuals per breed from Ningxiang, Duroc, and DLY). Cluster analysis was performed using the detected VFCs and their contents. The results show that Ningxiang pigs can be well separated from the two Western pig breeds and that Duroc and DLY pigs are similar and have an intersection (Figure 4). The results of the PCA are consistent with expectations since DLY and Duroc pigs are more closely connected by blood, because Duroc is DLY’s male parent. The two foreign pigs and the Ningxiang pig are distant cousins. Expectations also state that the clustering outcomes should be different from the two Western pig breeds. Therefore, we believe that this experimental method is feasible and representative for the detection of volatile flavor substances in boiled pork.

## 4. Conclusions

In this study, a HS-SPME-GC-MS method for the detection of VFCs in cooked pork was optimized. First, a weak polar chromatographic column DB-5-60 m can be used to separate more VFCs from cooked pork. Second, the optimized detection conditions are as follows: weigh 5 g meat sample, homogenize it in a homogenizer for 30 s, heat it to 100 °C for 30 min, balance it at 70 °C for 30 min, and then extract it at 100 °C for 50 min with a Gray 50/30 extraction head, which enhances the ability to extract and separate VFCs from cooked pork. In addition, the optimized experimental method was used to detect VFCs in Ningxiang, Duroc, and DLY pigs and analyze the principal components of the three pig breeds. It is not difficult to conclude that the optimized pretreatment method is able to distinguish the three pig breeds well, which also indicates that the method is feasible and representative. The sensory impression of the VFCs of pork comes mainly after cooking. So, it becomes crucial to standardize the pretreatment conditions to detect VFCs in cooked pork, as well as providing reliability for the comparative analysis of the study results.

## Figures and Tables

**Table 1:** Extraction head information.

Extractor Model	Film Thickness	Coating Material	Manufacturer	Product Number
Grey50/30	50 μm/30 mm	DVB/CAR-PDMS	Supelco, Bellefonte, PA, USA	SAAB-57324U
Black75	75 μm	CAR/PDMS	Supelco, Bellefonte, PA, USA	SAAB-57318
Red100	100 μm	PDMS	Supelco, Bellefonte, PA, USA	SAAB-57300-U
Blue65	65 μm	PDMS/DVB	Supelco, Bellefonte, PA, USA	SAAB-57318

**Table 2:** Orthogonal test condition setting table.

	Heat Treatment Time	Equilibrium Time	Equilibrium Temperature	Extraction Time	Extraction Temperature
1	30 min	20 min	70 °C	40 min	70 °C
2	40 min	30 min	80 °C	50 min	80 °C
3	50 min	40 min	100 °C	60 min	100 °C

**Table 3:** Comparison of column results.

Extractor Model	Number of Peaks	Total Peak Area
DB-5-30 m	44	14,496,024
DB-5-60 m	67	26,231,349
HP-88-100 m	23	4,366,547

**Table 4:** HS-SPEM-GC-MS range analysis.

	Test Level	Heat Treatment Time	Equilibrium Time	Equilibrium Temperature	Extraction Time	Extraction Temperature
Total amount of substances	K1	76.83	75.25	77.18	72.92	73.58
	K2	73.73	79.5	78.83	82.82	77.58
	K3	77.75	75.18	74.08	74.83	79.09
	R	4.02	4.32	4.75	9.90	5.51
Total peak area	K1	3.74 × 10^7^	3.85 × 10^7^	4.10 × 10^7^	3.36 × 10^7^	3.41 × 10^7^
	K2	3.74 × 10^7^	3.79 × 10^7^	3.65 × 10^7^	4.21 × 10^7^	3.57 × 10^7^
	K3	4.01 × 10^7^	3.99 × 10^7^	3.89 × 10^7^	4.07 × 10^7^	4.70 × 10^7^
	R	2.72 × 10^7^	1.93 × 10^7^	4.4 × 10^7^	8.54 × 10^6^	1.29 × 10^7^
Alcohols’ number	K1	13.17	13.67	13.73	13.25	14.42
	K2	13.45	13.75	13.58	15.73	13.33
	K3	14.50	14.00	14.08	12.58	13.64
	R	1.33	0.33	0.50	3.14	1.08
Ketones’ number	K1	3.00	3.67	2.91	3.42	4.08
	K2	4.09	3.58	4.42	4.55	4
	K3	4.25	4.36	4.17	3.67	3.45
	R	1.25	0.78	1.51	1.13	0.63
Aldehydes’ number	K1	29.08	30.33	29.64	28.67	28.92
	K2	28.18	28.5	29.58	30.55	29.58
	K3	29.17	28.73	28.42	28.50	29.09
	R	0.98	1.83	1.22	2.05	0.67
Aldehyde total peak area	K1	2.564 × 10^7^	2.606 × 10^7^	2.803 × 10^7^	2.235 × 10^7^	2.170 × 10^7^
	K2	2.544 × 10^7^	2.582 × 10^7^	2.407 × 10^7^	2.821 × 10^7^	2.408 × 10^7^
	K3	2.645 × 10^7^	2.679 × 10^7^	2.668 × 10^7^	2.823 × 10^7^	3.344 × 10^7^
	R	1.007 × 10^6^	9.721 × 10^5^	3.964 × 10^6^	5.887 × 10^6^	1.173 × 10^7^

## Data Availability

The data presented in this study are available from the corresponding authors upon request.

## Supplementary Materials

The following supporting information can be downloaded at: https://www.mdpi.com/article/10.3390/foods12020297/s1, Figure S1: Total ion chromatograms of the three Capillary columns; Figure S2. Total ion chromatograms of the four extraction heads; Figure S3. Total ion chromatograms at different heat treatment time; Figure S4. Total ion chromatograms at different equilibrium times; Figure S5. Total ion chromatograms at different equilibrium temperatures; Figure S6. Total ion chromatograms at different extraction times; Figure S7. Total ion chromatograms at different extraction temperatures.
