# Supplementary material for: Optimization of HS-SPME-GC-MS for the Determination of Volatile Flavor Compounds in Ningxiang Pork
Source: Foods. 2023 Jan 8;12(2):297. doi: 10.3390/foods12020297 (PMC9857953; doi:10.3390/foods12020297)

Figure S1. Total ion chromatograms of the three Capillary columns.

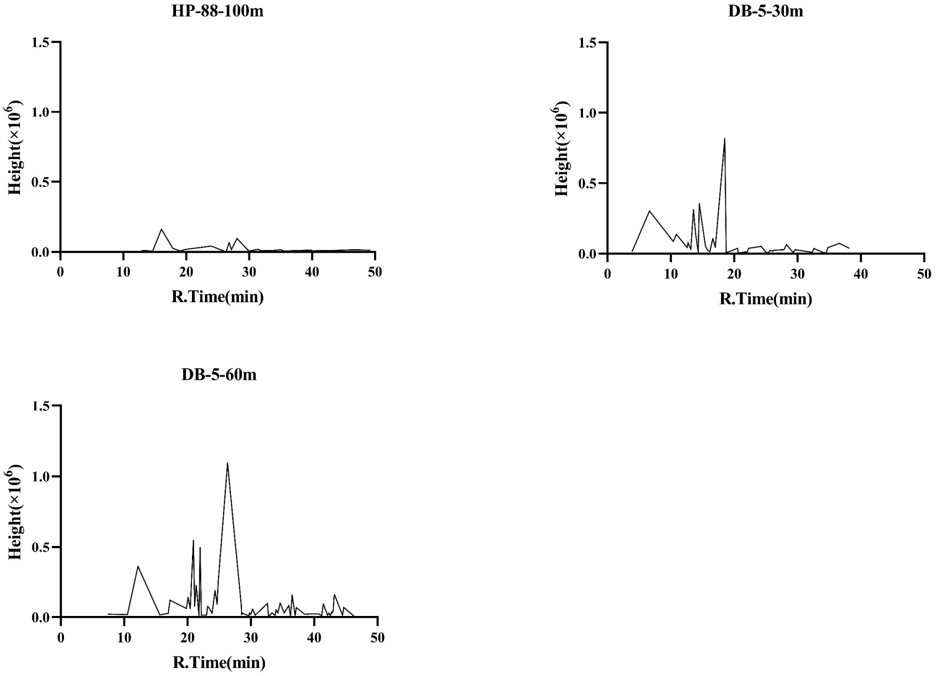

Figure S2. Total ion chromatograms of the four extraction heads

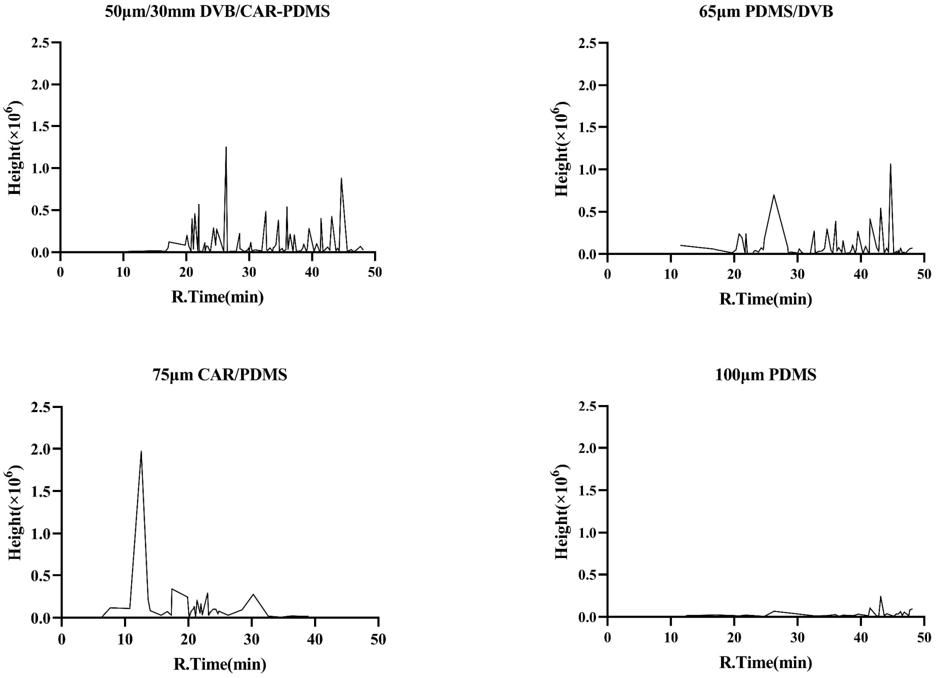

Figure S3. Total ion chromatograms at different heat treatment time

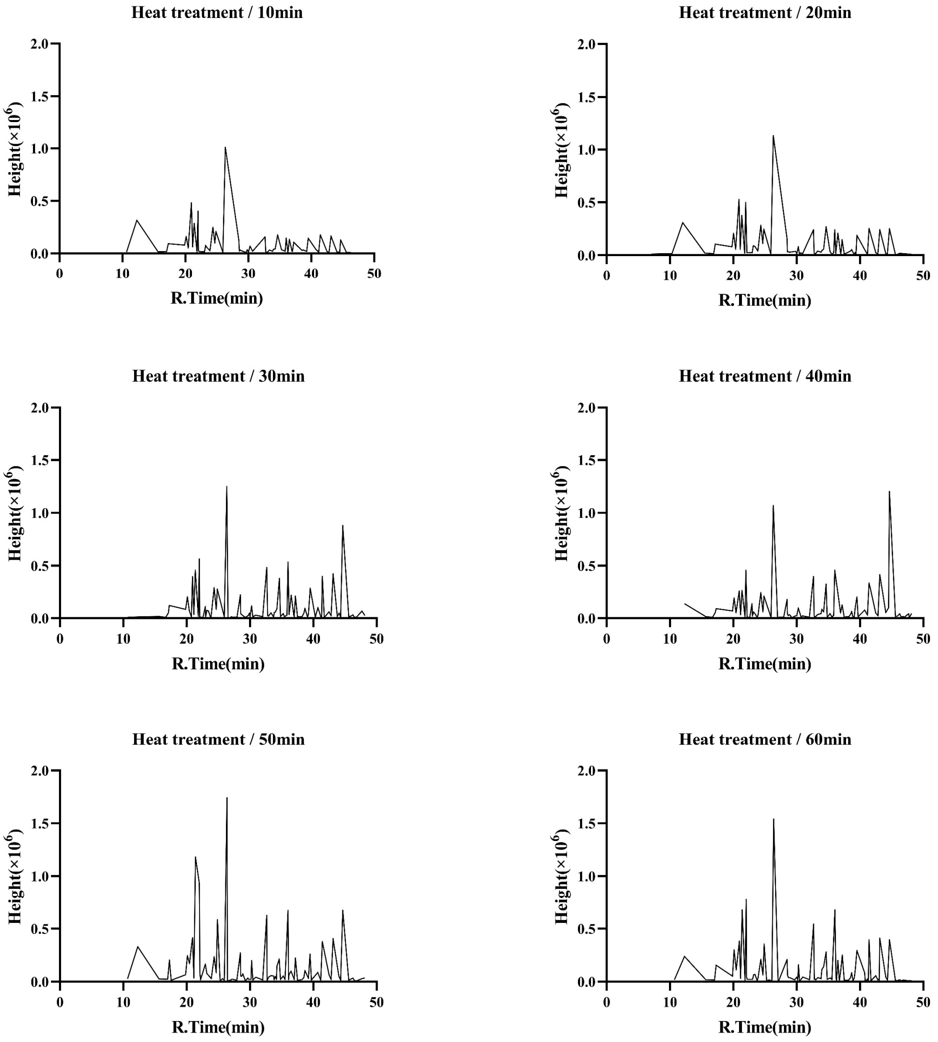

Figure S4. Total ion chromatograms at different equilibrium times

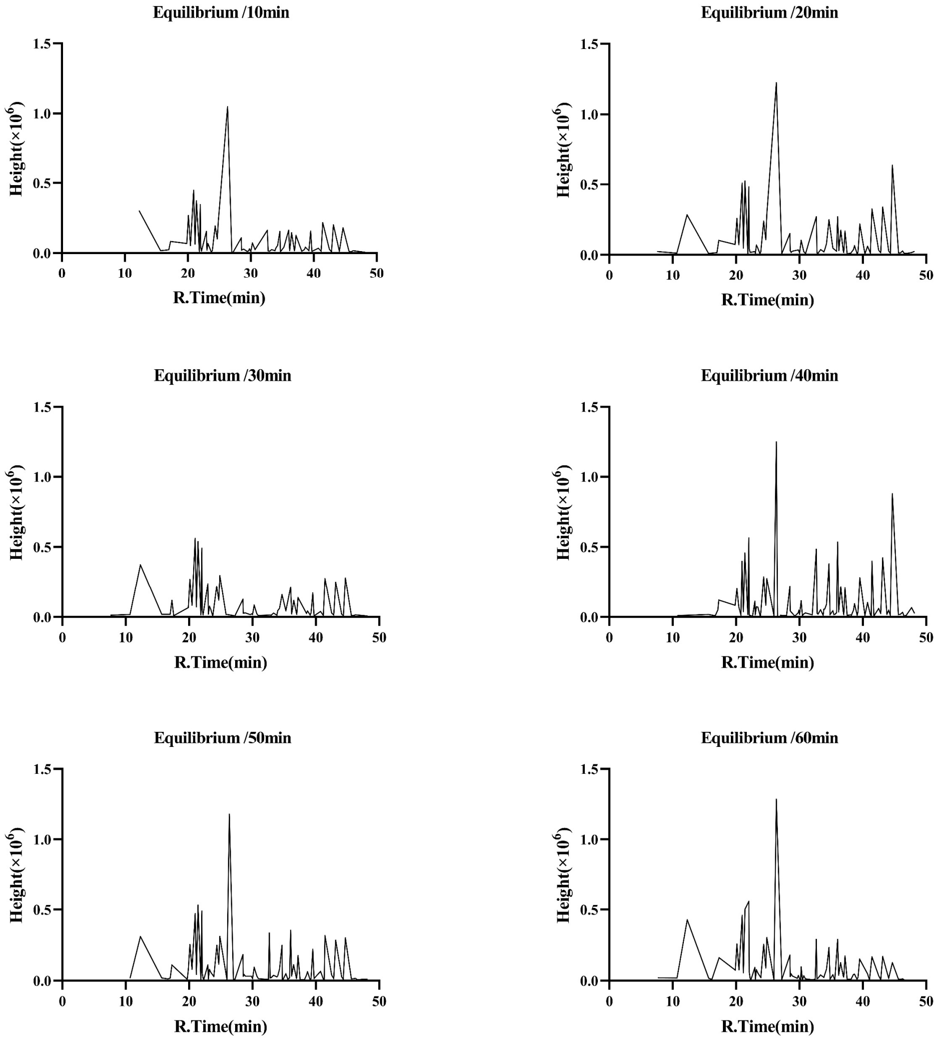

Figure S5. Total ion chromatograms at different equilibrium temperatures

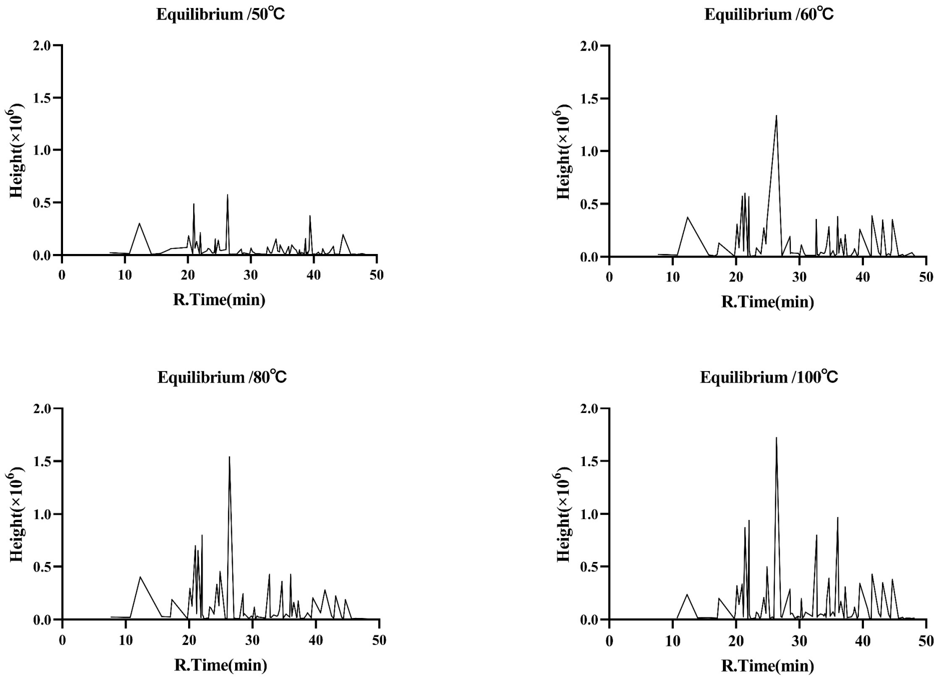

Figure S6. Total ion chromatograms at different extraction times

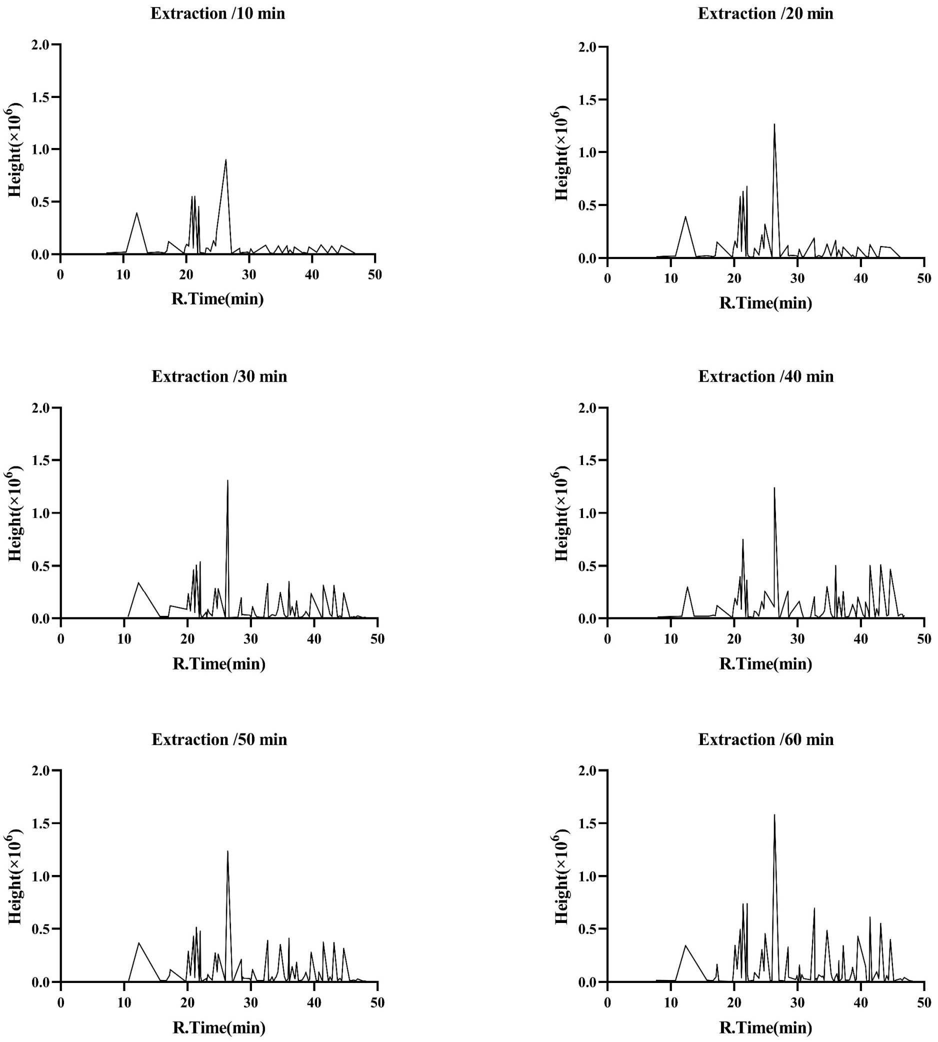

Figure S7. Total ion chromatograms at different extraction temperatures

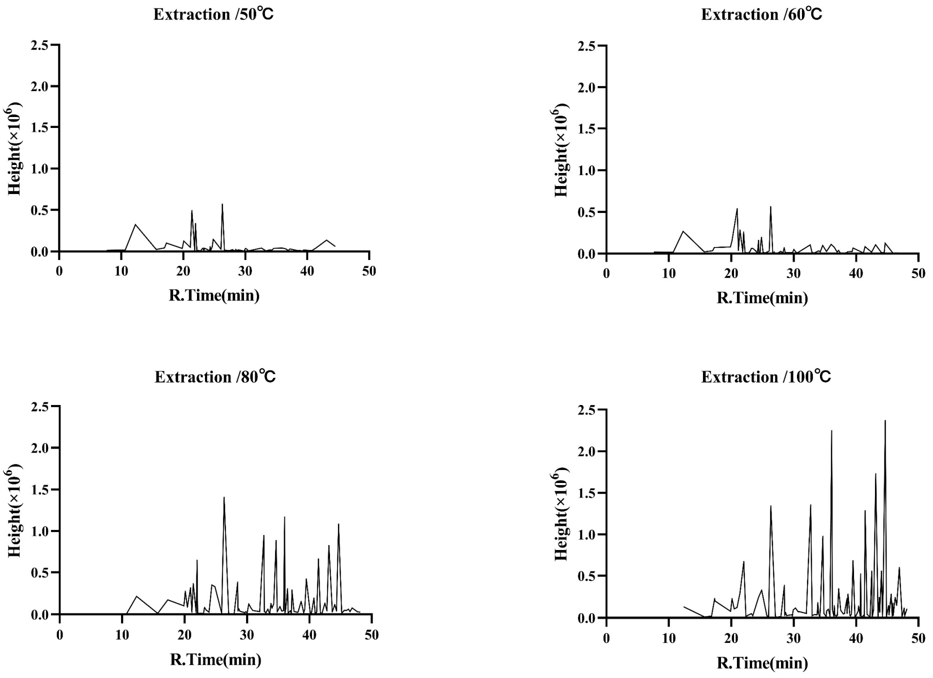

Supplement: Supplementary file 1 [file foods-12-00297-s001.zip › foods-2059606-supplementary.pdf]
